# Bioinformatics Education—Perspectives and Challenges out of Africa

**DOI:** 10.1093/bib/bbu022

**Published:** 2014-07-02

**Authors:** Özlem Tastan Bishop, Ezekiel F. Adebiyi, Ahmed M. Alzohairy, Dean Everett, Kais Ghedira, Amel Ghouila, Judit Kumuthini, Nicola J. Mulder, Sumir Panji, Hugh-G. Patterton, (for the H3ABioNet Consortium, as members of The H3Africa Consortium)

**Keywords:** bioinformatics education, bioinformatics in Africa, postgraduate program

## Abstract

The discipline of bioinformatics has developed rapidly since the complete sequencing of the first genomes in the 1990s. The development of many high-throughput techniques during the last decades has ensured that bioinformatics has grown into a discipline that overlaps with, and is required for, the modern practice of virtually every field in the life sciences. This has placed a scientific premium on the availability of skilled bioinformaticians, a qualification that is extremely scarce on the African continent. The reasons for this are numerous, although the absence of a skilled bioinformatician at academic institutions to initiate a training process and build sustained capacity seems to be a common African shortcoming. This dearth of bioinformatics expertise has had a knock-on effect on the establishment of many modern high-throughput projects at African institutes, including the comprehensive and systematic analysis of genomes from African populations, which are among the most genetically diverse anywhere on the planet. Recent funding initiatives from the National Institutes of Health and the Wellcome Trust are aimed at ameliorating this shortcoming. In this paper, we discuss the problems that have limited the establishment of the bioinformatics field in Africa, as well as propose specific actions that will help with the education and training of bioinformaticians on the continent. This is an absolute requirement in anticipation of a boom in high-throughput approaches to human health issues unique to data from African populations.

## INTRODUCTION

The field of bioinformatics is increasingly dependent on the effective interpretation of large and complex sets of data. The requisite skills rest predominantly with researchers in developed countries versed in the use of sophisticated computational tools and advanced statistical tests. In Africa, the relevant skills are not readily available, and are therefore frequently sought outside Africa. Current development capacity in bioinformatics is largely based on input from a few isolated senior scientists in Africa and abroad. To address this, a number of initiatives from the National Institutes of Health (NIH, http://www.nih.gov/) and the Wellcome Trust (http://www.wellcome.ac.uk/) are underway to strengthen research capacity in genomics across Africa, including bioinformatics as a key area. The Human Heredity and Health in Africa (H3Africa, www.h3africa.org/) initiative [1] and Pan African Bioinformatics Network for H3Africa (H3ABioNet, www.h3abionet.org/) [2] as part of H3Africa Consortium hold great promise for setting up sustainable and active bioinformatics training initiatives and collaborations on the African continent, in support of genomics initiatives, aimed at both the African genetic richness, as well as the study of genomic links to an African predisposition for specific diseases.

This article provides an overview of the development of bioinformatics as a discipline and bioinformatics education from an African perspective.

## DEVELOPMENT OF BIOINFORMATICS AS A DISCIPLINE

### In general

The term ‘bioinformatics’ was first used in 1989 by Daniel R. Masys [3]; however, bioinformatics research can be traced back as early as the 1960s [4]. Since then, bioinformatics has evolved drastically. However, the evolution of bioinformatics in the last almost 50 years has not occurred in a linear fashion, but rather in stages, and has been closely linked to advances in both life science and computer science.

The 1970s and the 1980s were the decades during which protein and nucleic acid sequence databases were established, and the increasing need for the retrieval and analysis of data in the databases led to the development of accompanying tools. Yet, the dissemination of data and the use of these tools were daunting to many, since the operating systems and computer programs were not user-friendly [5]. The emergence of the Human Genome Project made 1985 and 1986 particularly important years in bioinformatics history, but more significantly, the development of web interfaces and increasing connectivity to the Internet in the 1990s started to make the data more accessible. Meantime, in 1995, the first genome sequence of a free living organism, *Haemophilus influenza*, was completed [6]. The first draft of the human genome sequence was published in 2001 [7], and the advancement of sequencing technologies during the preceding 16 years of the project duration was beyond anyone’s imagination.

In the past decade, the advancement of modern high-throughput techniques in life sciences, coupled with developments in computing technology, opened a new era for bioinformatics, which has rapidly grown into a discipline. Today, the scale of biological data available to organize, analyse and disseminate is daunting, yet offers exciting possibilities that could provide answers to many central questions in life sciences research.

### In Africa

**Figure 1:**
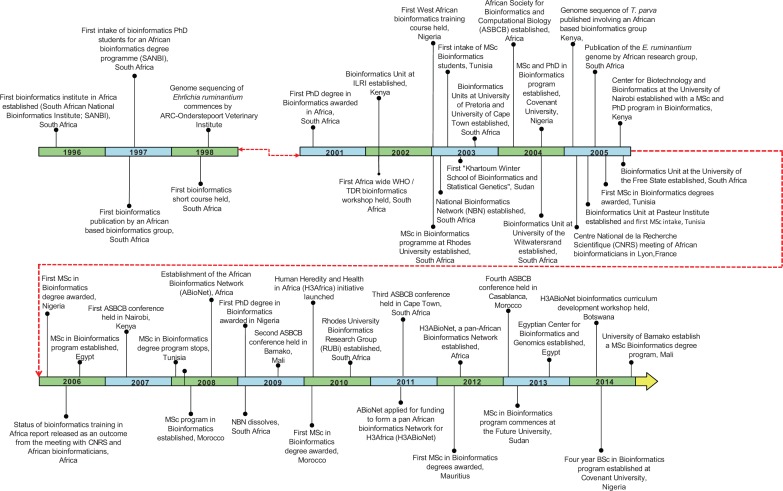
The timeline of bioinformatics development in Africa showing selected key events.

A summary of some key events in the development of bioinformatics in Africa is given in Figure 1, with further details supplied in Supplementary Data S1. The first event was the establishment of the South Africa National Bioinformatics Institute (SANBI, http://www.sanbi.ac.za/) at the University of the Western Cape, Cape Town, in 1996. This was followed in the early 2000s, by discussions in South Africa that led to the establishment of the National Bioinformatics Network (NBN). The NBN established a central office and regional nodes at several South African universities. During its existence, NBN provided a 6-month training program for South African postgraduate bioinformatics students. Also, within this period, the WHO sponsored bioinformatics training courses at SANBI, starting from 2001, for African researchers. The basis for the African Bioinformatics Network (ABioNet)—an initiative of trainers and researchers in bioinformatics across Africa—was started in 2002, and formally initiated in 2008 in Abuja, Nigeria. The establishment of the African Society for Bioinformatics and Computational Biology (ASBCB, http://www.asbcb.org/) originated from a WHO/Tropical Disease Research (TDR) workshop in February, 2004. Between 2003 and 2005, a number of individuals across other African institutions led to the further development of bioinformatics in Africa. A pivotal moment in African bioinformatics came in 2005 when the Centre National de la Recherche Scientifique brought together African Scientists working in the field of bioinformatics to a meeting in Lyon, France (http://www.lirmm.fr/france_afrique/). Consequently, the ASBCB began running conferences on the bioinformatics of disease vectors, every two years. The first one was held in Nairobi in 2007. Subsequent meetings were organized in partnership with the International Society of Computational Biology (ISCB, http://www.iscb.org/) (Bamako, Mali in 2009; Cape Town, South Africa in 2011; Casablanca, Morocco in 2013). Student communities associated with ASBCB have since self-organized into African regional student groups (RSGs), affiliated with ISCB. The RSGs organized training workshops and collaborated in hosting a Virtual Bioinformatics Conference, African Virtual Conference on Bioinformatics (AFBIX’09) [8]. The next pivotal event occurred in 2008. A WHO-sponsored meeting took place in Abuja, Nigeria, in April 2008, where it was proposed that ABioNet should develop African bioinformatics based on a system of collaborating nodes. Responding to the challenge of ensuring that human genomics research in Africa was truly an African endeavour, the African Society of Human Genetics (http://www.afshg.org/), the NIH and the Wellcome Trust convened the Frontiers Meeting in Yaoundé, Cameroon, in March 2009, to develop a research agenda to study genetic diversity in health and disease in African populations. Following the Cameroon meeting, the concept of H3Africa was developed, and the initiative, with substantial funding from both the NIH Common Fund and the Wellcome Trust, was announced in 2010. Within the scope of the H3Africa initiative, the previously unfunded ABioNet was successful in securing substantial NIH funding, transforming into H3ABioNet. The *raison d'etre *of H3ABioNet is to provide the required bioinformatics support to projects addressing human health and heredity in Africa in the H3Africa initiative. Importantly, the H3ABioNet network grew largely from existing continent-wide organizations already involved in bioinformatics. Now H3ABioNet currently comprises 32 nodes across the continent [1, 2].

## DEVELOPMENT OF BIOINFORMATICS EDUCATION

### In general

Bioinformatics is a relatively new discipline, yet bioinformatics education is even more recent, as the first education paper was written only 16 years ago [9]. The complexity and amount of the biological data produced, primarily by genome sequencing projects, and subsequently by other high-throughput approaches, caused an urgent need, both from academia and industry, for individuals skilled in bioinformatics. Since then, owing to its interdisciplinary nature, the successful implementation of bioinformatics education has been a matter of ongoing discussion. For example, ‘what is an appropriate curriculum?’ is an issue that has been continually raised from the start. It has become clear that an important factor in deciding on a curriculum is the target, e.g. training life scientists to use certain bioinformatics tools or training professional bioinformaticians.

Early attempts focused on establishing short training programs for available graduates in related disciplines. This was followed by formal education programs, mainly at postgraduate level, but also some at the undergraduate level, initiated by various universities and institutions worldwide [10–13]. Today, the majority of bioinformatics teaching is at the postgraduate level, and students tend to enroll in these programs with different backgrounds in terms of formal training received. This has, of course, its own challenges from both lecturer and student perspectives. For the lecturer, the main difficulty is how to handle such a diversity of prior knowledge (e.g. large variations in levels of computing skills or limited exposure to biological concepts). The students themselves also obviously suffer from the fact that their peers are from diverse backgrounds, so the lectures might not be pitched ideally to their own backgrounds.

This and a number of other common challenges led to further discussions on how to handle education in bioinformatics. In 2001, the Education Committee of the ISCB (http://www.iscb.org/iscb-education-committee) produced a document suggesting core and supporting contents of bioinformatics programs [14]*.* Bioinformatics education received further formal recognition in 2005, when the ISCB, for the first time, included bioinformatics education as a mainstream session at its annual conference [14]. The ISCB Education Committee created a Curriculum Task Force in 2011 and this Task Force has recently published two reports [15, 16]. Contributions from ISCB also include a database of bioinformatics degree programs throughout the world (http://www.iscb.org/iscb-degree-certificate-programs). Another recent event is the establishment of the Global Organisation for Bioinformatics Learning, Education and Training (GOBLET, www.mygoblet.org) [17], which aims to provide a global support network for bioinformatics training. In Africa, H3ABioNet held a bioinformatics degree development workshop in Botswana recently, leading to the establishment of an African Bioinformatics Education Committee, which plans to work with the ISCB and GOBLET on curriculum development. These are by no means the only bioinformatics education initiatives: most bioinformatics societies or regional networks have some bioinformatics training activities, for example, but reviewing these is beyond the scope of this article.

### In Africa

Bioinformatics education in Africa is currently being revitalized through initiatives such as H3ABioNet. One of the main remits of the network is to build human capacity in bioinformatics in Africa, which involves compiling information on existing degree programs and courses on the continent and working towards development of new ones where there are gaps. There are already a number of formal bioinformatics programs established at African institutions [18], some of which are presented in Table 1. Further, many institutions are in the process of setting up local programs. All the African countries sampled that do not have a bioinformatics degree have expressed interest in devising a curriculum and a degree program in bioinformatics, motivated by falling costs of biological data generation, and the dearth of human capital to organize, investigate and make sense of this deluge of data.

**Table 1:** Bioinformatics degree programs offered in African countries

Country	University	Degree Program	URL
Egypt	Ain Shams University	Dp, BSc, MSc (structured)	http://cis.asu.edu.eg//english/article.php?action=show&id=6169
	Helwan University		http://www.helwan.edu.eg/fcih/index.php/en/2012–12–15–18–57–14/79-computer-science/103-the-department-word
Kenya	Jomo Kenyatta University	MSc (structured)	http://www.jkuat.ac.ke/postgraduate-courses/msc-molecular-biology-bioinformatics/
	University of Nairobi		http://cebib.uonbi.ac.ke//uon_degrees_details/264. reg_anchor_264_271
Mali	University of Bamako	MSc (structured)	
Mauritius	University of Mauritius	MSc (structured), PhD	http://www.uom.ac.mu/programmes/Courses/FOA/YR2013/Postgraduate/PDF/A517.pdf
Nigeria	Covenant University	BSc, MSc (structured), PhD	http://cis.covenantuniversity.edu.ng/Programmes/Postgraduate
South Africa	Rhodes University	MSc (structured)	http://rubi.ru.ac.za/, also see [18]
	University of Cape Town	Hons, MSc, PhD	http://www.cbio.uct.ac.za/
	University of Pretoria	Hons, MSc, PhD	http://web.up.ac.za/default.asp?ipkCategoryID=21675
	University of Western Cape	MSc, PhD	http://www.sanbi.ac.za
	University of Witwaterstrand	MSc, PhD	http://www.bioinf.wits.ac.za/Bioinformatics/Home_page.html
Sudan	Future University of Sudan	BSc, MSc, PhD	http://www.futureu.edu.sd/fupage.php?pgt=1&facid=4&id=120

The data presented comes from responses to a survey, and so may not be complete. Structured MSc includes a combination of course work and research project.

The available programs are not enough to handle the demand, and South African Bioinformatics groups are overwhelmed with applicants from many other African countries [18]. The main reason is most likely the lack of academic faculty able to supervise purely bioinformatics research projects in some of the African institutions.

It is important to distinguish between formal qualifications in bioinformatics (with a specific bioinformatics course code), and degrees in related subjects where the projects have a bioinformatics component or focus, e.g. a Masters or PhD in computer science or biochemistry. From the undergraduate perspective, it is mostly the case that traditional BSc degrees are offered, some of which may offer modules in bioinformatics. Many discussions have been held on whether a focus should be placed on undergraduate training in bioinformatics, but even where bioinformatics undergraduate degrees are offered, the corresponding Masters and PhD programs are still overwhelmingly filled with students who did not come through the undergraduate stream.

Most of the bioinformatics programs offered at Masters level have a component of course work and lectures to accommodate students with different educational backgrounds. These Masters courses cover introductory programming and molecular biology, before moving to more advanced topics that are based on the area of expertise that is available at that institution. However, there is no unified Masters bioinformatics curriculum within Africa.

Many PhD degrees are mainly obtained by research into specific topics that require a significant portion to be devoted to bioinformatics under a supervisor/co-supervisor who is an established bioinformatician. In some cases, various African research institutes have formed collaborations with established bioinformatics departments and have their students enrolled at those universities while conducting their research at their home institute to allow the transfer of skills and technology between the partner institutions.

In addition to degree programs, many short training workshops are offered throughout Africa, up until recently, mostly organized and sponsored by foreign institutions. Generally, workshops are targeted at a variety of groups, including bioinformaticians and wet-lab researchers. An example is the Wellcome Trust that offers courses in Africa every year (see Supplementary Data S1).

Through networks such as H3ABioNet and ASBCB, we have been able to track some of the existing bioinformatics students in Africa and the progress of their careers. ASBCB runs an African bioinformatics conference every two years with associated workshops, and there is usually a large overlap in participants from year to year. However, we are starting to see an increase in the number of students attending the conference, which is encouraging and may be a reflection of the increased interest in bioinformatics in Africa, and possibly also an increase in the number of workshops that are offered.

## CHALLENGES AND POSSIBLE SOLUTIONS IN AFRICAN CONTEXT

There are many challenges faced in bioinformatics education that are commonly encountered throughout the world, for instance, the diversity in academic background of students (see Section 3); and the breadth of the discipline, ranging from life sciences through computer science to statistics. Furthermore, bioinformatics is a fast-evolving discipline that requires bioinformatics researchers/lecturers to continually keep abreast of new developments and learn new skills. However, such matters are not discussed here. The focus of this section is, rather, on two major issues hindering research on modern high-throughput genomics and bioinformatics projects in African institutions: shortage of trained bioinformaticians and infrastructural problems. Here, we discuss these obstacles with some proposed solutions resulting from our experiences in the H3ABioNet network.

### Building training capacity

*Continuing mentorship and support:* Short training courses are important to familiarize students with various aspects of bioinformatics. However, once students leave the course, they are usually on their own. It is important to follow-up to ensure that students can consolidate and put into practice what they have learned. A system of continuing mentorship and support would ensure that students are able to have regular contact with trainers. ASBCB and now also H3ABioNet have been trying to implement a mentorship program for students who do not have access to experienced faculty in their own institution. It has been difficult to maintain commitment from mentors abroad, but we have had success in some instances with mentors or co-supervisors at other African institutions. This initiative needs to be further developed by having students participate in laboratory meetings via Skype, for example, as regular communication is key to making this successful. H3ABioNet is also training students through involvement in joint research projects relevant to the goals of the network. Several cross-institution projects have been initiated, some of which are being implemented through internships (see below) or joint supervision from a distance. More experienced bioinformatics nodes within H3ABioNet also provide mentoring in terms of training materials, access to trainers and assistance with the logistics of organizing training workshops for less-developed nodes.

*Train-the-trainers: *Train-the-trainer is typically an intense course to get a lecturer up to speed and place that lecturer in a position to train students. It aims to keep lecturers updated in this dynamic field. This would typically be someone with little training in bioinformatics, but with an interest and talent for the area. Train-the-trainer courses might go hand-in-hand with providing material for training at home institutions as well as additional support to deal with questions or difficulties when the trained trainer presents the course at their home institution. Train-the-trainer courses are also a cost-saving option, especially in African countries, when a group from the same laboratory or institution needs to be trained in a specific topic or software. In 2013, H3ABioNet ran a 3-week train-the-trainer course in Kenya, training young staff members in a number of bioinformatics topics [Python programming, biostatistics, next-generation sequencing (NGS) and genome-wide associations study (GWAS) analyses]. A total of 20 participants attended the course, several of which went on to teach the same course back at their home institutions. Others were not yet comfortable with their expertise in the area, so follow-up is being done, and these trainees will be given an opportunity to be teaching assistants at upcoming courses until they are confident to teach themselves.

*Internship programs:* Internship programs are designed to train students in a specific area of bioinformatics. Students from African institutions visit bioinformatics laboratory at other African institutions for few months to work on a specific project. They are expected to disseminate what was learnt on return to the home institution. Such a program is provided by H3ABioNet, where interns are placed at host institutions with which they have initiated a joint research project, or where they would like to learn a bioinformatics technique. Internships focus on real data from research projects, making the internship useful in progressing the projects at the same time as building skills. In 2014, six student interns were funded, with one student from Tunisia, one from Botswana and two from Tanzania placed at bioinformatics laboratories in South Africa, one student from Tunisia hosted by the University of Illinois and one from Nigeria hosted by a laboratory in Germany.

*Knowledge transfer via visiting scientists:* The idea is that a visiting scientist would be hosted in Africa for a period of 2–6 months, and work together with African researchers on a problem of mutual interest. This ‘on-the-project’ training solidifies knowledge transfer. Some universities have special funding for this purpose, and one example of such a program specifically for bioinformatics is offered by Centre for Proteomic and Genomic Research (http://ktp.cpgr.org.za/).

*E-learning:* A number of online platforms have emerged all over the world [19–22] impacting on education across many fields [23]; see [24] for a discussion of the terminologies used in this context. E-learning platforms provide materials and computer-assisted courses that involve hands-on practical exercises [25, 26]. In the bioinformatics field, opportunities vary from training in the use of specific tools (for example, as offered by the European Bioinformatics Institute (http://www.ebi.ac.uk/training/online/)), to accredited online bioinformatics Masters degree programs (for example, as offered by Johns Hopkins University (http://advanced.jhu.edu/academics/graduate-degree-programs/bioinformatics/)). Recently, new e-learning initiatives have emerged in African countries [21, 22]. Although e-learning can be efficient and cost-effective, a drawback is the time and resources needed to set up the study material, as well as the need for computers and a good Internet connection, which is problematic in parts of Africa. A solution to this is to provide complete course materials on a portable device that is independent of the Internet.

### Lack of infrastructure

One of the key challenges within the African context is reliable access to the Internet, as many bioinformatics tools are web-based and even client tools often require access to online datasets [It also should be noted that while Internet performance is presently problematic in many places in Africa problematic at present, this is not uniformly so and the situation is changing (Table 2)]. The eBioKits initiative (http://www.ebiokit.eu/) tackles this challenge: it houses popular biological databases and tools such as Ensembl, NCBI-BLAST, EMBOSS, PLINK and functions as an affordable low-cost server by enabling computers to connect via the local area network and run various bioinformatics applications. The eBioKits initiative was specifically borne to overcome the challenge of poor Internet connectivity in Africa. As the eBioKits mitigates the need for connecting to external databases and has a variety of self-contained tutorials, it has been successfully used in various training workshops within Africa. The eBioKits also offers small research groups a platform to engage with bioinformatics in their research [27]. H3ABioNet has provided a significant number of eBioKits throughout its African network in conjunction with training workshops.

**Table 2:** Internet performance in Africa

Internet penetration rate Top 10 countries	Users (in millions)	Fastest broadband speed Top 10 countries	Mbps; World ranking
Nigeria	48.4	Ghana	5.14; 73^rd^
Egypt	29.8	Kenya	4.94; 75^th^
Morocco	16.5	Angola	4.53; 80^th^
Kenya	12	Rwanda	3.28; 97^th^
South Africa	8.5	Zimbabwe	2.98; 104^th^
Sudan	6.5	South Africa	2.98; 105^th^
Tanzania	5.6	Libya	2.94; 107^th^
Algeria	5.2	Morocco	2.77; 109^th^
Uganda	4.4	Nigeria	2.30; 129^th^
Tunisia	4.2	Tunisia	2.12; 135^th^

Data gathered from The Internet World Stats (http://www.internetworldstats.com/) (The Miniwatts Marketing Group, 2012), and Ookla’s NetIndex (http://www.netindex.com/) in June and March 2012, respectively.

Some bioinformatics groups in Africa that do provide training usually have a local server. Unless these servers are powerful and can handle numerous workshop participants processing data simultaneously, a commonly used approach is to subset the data sets used during the training. This is particularly effective when providing bioinformatics training pertaining to NGS data where the mapping of reads and variant calling is done using a single chromosome as opposed to whole genomes.

Another key challenge to course organization is training infrastructure, unless the host has a dedicated computer training laboratory. University computer laboratories are generally shared resources prioritized for undergraduate courses, are highly subscribed and are in short supply. As these computer training laboratories are used by a variety of disciplines, and are generally administered by a university’s IT department, the computers are usually running fixed lists of Windows software packages with little flexibility to provide a Linux-based environment. One solution is to boot off a flash memory stick, and present the user with a fully functioning Linux environment with the pre-requisite bioinformatics tools [28]. The user can write the results, files and computer programs created during the workshop to the memory stick for access post-workshop. A current limiting factor, however, is that the physical memory is inadequate when dealing with NGS datasets. The maturity of virtualization technology has managed to sidestep the above limitations [29]. The creation and deployment of virtual machines (VM) is increasingly being used for bioinformatics workshops within Africa because of the advantages of being able to create and use the same VM configuration on multiple computers, and also make the VM available to participants post-workshop. Projects like H3ABioNet have helped to renovate and furnish various training labs with computers and servers within the network in Africa to be used for bioinformatics training and research within that region, helping to alleviate some of the accessibility to infrastructure dilemmas encountered.

Other technologies that are becoming more commonly used in bioinformatics NGS and Galaxy training within Africa are cloud-based such as Amazon web services, which will become more widespread as accessibility to the Internet inevitably improves. Another exciting technology currently being used within Africa and H3ABioNet to overcome the vast geographical distances is the use of live video streaming services such as Vidyo. This enables geographically different classrooms to participate in a training workshop and has the advantage of being live and interactive. H3ABioNet has used the system to provide the train-the-trainers workshop held at International Centre of Insect Physiology and Ecology (ICIPE), Kenya, to classrooms in Nigeria and Tunisia, simultaneously in 2013. The Vidyo system has also been used successfully to provide a training module in introduction to bioinformatics resources by a trainer from the University of the Witwatersrand Bioinformatics unit in Johannesburg, South Africa, to a classroom of postgraduate students at the Covenant University Bioinformatics Research Unit in Ota, Nigeria. Apart from cost and technical expertise required, a drawback of using a system like Vidyo is the need for a strong cadre of training assistants to be present at the classroom location to assist participants with the practical components. However, we envision that these training assistants will gain more practical training experience and would ultimately be able to conduct the training workshops

## CONCLUSION

African institutes have made great strides in establishing bioinformatics as a subject in undergraduate curricula and as a postgraduate field. Significantly, the establishment of ASBCB and ABioNet has played a crucial role in providing a critical mass of expertise that facilitated the establishment and nurturing of such training programs. However, despite these energetic efforts, there is still a significant shortage of trained bioinformaticians in Africa. This is partly because of insufficient infrastructure but, critically, also because of the absence of suitably qualified lecturers and academic mentors. The absence of lecturers with an appropriate and up-to-date skill set has caused the absence of bioinformatics training courses at many institutes, and has had a severely retarding effect on the undertaking of modern, high-throughput projects in Africa. This has also been a particular impediment to genomic studies related to the rich genetic diversity of African populations, and the systematic study of the possible genetic predisposition to specific diseases in Africa.

This article has proposed a number of approaches to overcome the shortage of trained bioinformaticians on the continent. In addition to train-the-trainer courses, mentorship, internship and knowledge transfer programs, which build capacity in bioinformatics at African institutes, there is also a need for refresher or follow-up courses, to ensure that skill sets are retained and current. E-learning platforms are an option that have been successfully implemented at some established institutes in the developed world, and is being considered for Africa.

Bioinformatics is not widely appreciated as a career choice, and thus there is limited demand for coherent undergraduate training in computer science and life sciences. Therefore, in postgraduate programs, students predominantly come from one of either of these backgrounds, and the development of a curriculum that is both appropriately pitched, and sufficiently substantial, remains challenging. The need for responsive and effective groupings to provide guidance to academic institutes to define training frameworks and content also underscores the extremely rapid evolution of the landscape of modern life sciences. This is something that institutes must be aware of, and respond to, to remain relevant in research and training in life sciences.

The genomics initiatives funded by the NIH and the Wellcome Trust in Africa, clearly focused attention on the crucial and central need for bioinformatics. There are many extremely topical genomics projects relevant to Africa. These include identifying SNPs or wider mutations, including epigenomic modifications, linked to disease susceptibility or resistance in African populations. Modern man originated on the African continent, and the mapping of migrations on and from the continent from genomic data would provide fundamental insights into the development and relationship of global human settlements.

The biomedical industry is a pillar of socio-economic development in developed nations, and can be a catalyst of economic growth in Africa. However, an overwhelming number of African countries fall below the average standard indices of technology and science capacity [30]. In addition, over the years, Africa has witnessed a steady loss of university staff, leading to low scientific research output, and to poor preparation of the next generation of African biotechnology (biomedical) scientists and bioinformaticians. An enthusiastic adoption of bioinformatics as a discipline at African institutes must continue to be developed and nurtured to ensure that the genomics area, and all the medical and social benefits that it brings, does not pass Africa and its peoples by.

## SUPPLEMENTARY DATA

Supplementary data are available online at http://bib.oxfordjournals.org/.

**Key Points**

- The recent initiatives funded by the NIH and the Wellcome Trust, such as H3Africa Consortium and as a part of the Consortium, H3ABioNet, aim to strengthen research capacity in genomics across Africa including bioinformatics as a key area. Bioinformatics analysis of genomics data will provide fundamental insights into both the African genetic richness and the understanding of specific diseases.
- Development of bioinformatics field in Africa as a discipline has gained speed in the last 15 years: Its presence has grown from just one institution to at least 32 across Africa; and an African Society as well as various national societies, have been established.
- Bioinformatics education in Africa is currently being revitalized through initiatives such as H3ABioNet: One of the main remits of the network is to build human capacity in bioinformatics in Africa. There are already a number of formal bioinformatics programs established at African institutions. Further, many institutions are in the process of setting up local programs.
- The shortage of trained bioinformaticians on the continent is one of the main problems to the further development of bioinformatics: Proposed solutions include continuous mentorship and support, train-the-trainers, internships, knowledge transfer programs and e-learning platforms.

Supplementary Data
