# Supplementary material for: Bioinformatics Education—Perspectives and Challenges out of Africa
Source: Brief Bioinform. 2014 Jul 2;16(2):355–64. doi: 10.1093/bib/bbu022 (PMC4364068; doi:10.1093/bib/bbu022)
Supplement: Supplementary Data [file supp_16_2_355__index.html]

Bioinformatics Education—Perspectives and Challenges out of Africa — Bioinformatics Education—Perspectives and Challenges out of Africa — Supplementary Data 

# Bioinformatics Education—Perspectives and Challenges out of Africa

## Supplementary Data

files

**Files in this Data Supplement:**

- Supplementary Data - doc file
