# Supplementary material for: Bioinformatics Education—Perspectives and Challenges out of Africa
Source: Brief Bioinform. 2014 Jul 2;16(2):355–64. doi: 10.1093/bib/bbu022 (PMC4364068; doi:10.1093/bib/bbu022)
Supplement: Supplementary Data [file supp_bbu022_Supplementary_Data_1_FINAL_1April2014.doc]

**Supplementary Data 1:** Summary of the bioinformatics education and research related events in Africa.

**Year Event**

**1996** First bioinformatics institute in Africa (South African National Bioinformatics Institute -SANBI) was established in South Africa.

**1997** First intake of PhD in Bioinformatics students in Africa (SANBI, University of Western Cape).

**1997** First bioinformatics paper was published by an African based group (Hide et al. (1997). *Genome Informatics* pp187-196)

**1998** Genome sequencing of *Ehrlichia ruminantium* was commenced by Agriculture Research Council (ARC), Onderstepoort Veterinary Institute in Pretoria, South Africa.

**1999** University of KwaZulu-Natal (UKZN) Bioinformatics Unit was established.

**2001** First intake of MSc in Bioinformatics students in Africa (SANBI, UWC).

**2001** Sequencing of the *Theileria parva* genome by TIGR and the Institute for Livestock Research (ILRI), Kenya, commenced.

**2001** First online S* Star online bioinformatics course was held with SANBI providing a lecture on gene transcription and EST clustering. Of 96 students who completed the course, 9 were from Africa.

**2001** First PhD in Bioinformatics degree awarded in Africa (SANBI, UWC).

**2002** Bioinformatics Unit at Institute for Livestock Research (ILRI) was established in Nairobi, Kenya.

**2002** First Africa-wide WHO/TDR bioinformatics training workshop was held in South Africa (SANBI).

**2002** Establishment of a Bioinformatics service involved in education and research (CBS / Sfax), Tunisia

**2003** West African bioinformatics training course at the University of Ibadan, Nigeria was held.

**2003** Assembly and annotation of *Ehrlichia ruminantium* genome was commenced by Agriculture Research Council (ARC), Onderstepoort Veterinary Institute, Pretoria, South Africa.

**2003** MSc in Bioinformatics program in Tunisia commenced.

**2003** Bioinformatics Unit was established at the University of Cape Town, South Africa.

**2003** Bioinformatics Unit was established at the University of Pretoria, South Africa.

**2003** Second WHO/TDR bioinformatics training workshop held in South Africa (SANBI).

**2003** National Bioinformatics Network (NBN) was established in South Africa.

**2003** One year coursework and dissertation MSc Bioinformatics program was initiated at Rhodes University and run in 2003 and 2004.

**2003** First MSc in Bioinformatics degree awarded in Africa (SANBI / UWC).

**2003** University of Khartoum, Sudan held the first "Khartoum Winter School of Bioinformatics and Statistical Genetics".

**2004** African Society for Bioinformatics and Computational Biology (ASBCB) was established.

**2004** Bioinformatics Unit was established at the University of the Witwatersrand (WITS), South Africa

**2004** First NBN bioinformatics course was run, and repeated annually until 2009.

**2004** Third WHO/TDR bioinformatics training workshop was held in South Africa (SANBI).

**2004** Third and final West African bioinformatics workshop on malaria bioinformatics was held at University of Ibadan, Nigeria.

**2004** Symposium and workshop on bioinformatics and applied genomics, West African bioinformatics training course was held at University of Ibadan, Nigeria.

**2004** MSc and PhD degrees in Bioinformatics were established at University of Cape Town.

**2004** African Center for Training in Functional Genomics of Insect Vectors of Human Diseases was established in Bamako, Mali.

**2004** First WHO/TDR African training course on functional genomics of insect vectors of human diseases was held in Mali and repeated in 2005, 2006 and 2008.

**2004** MSc and PhD in Bioinformatics programs were established in Covenant University, Nigeria.

**2005** Centre National de la Recherche Scientifique (CNRS) meeting of African bioinformaticians in Lyon, France.

**2005** Centre for Biotechnology and Bioinformatics (CEBIB) was established at University of Nairobi, Kenya.

**2005** Bioinformatics Unit at Pasteur Institute, Tunisia was established.

**2005** First MSc in Bioinformatics degrees were awarded in Tunisia.

**2005** International Workshop on Pattern Discovery in Biology (IWPDB) at Covenent University, Nigeria – repeated in 2009

**2005** Genome sequence of *Theileria parva* was published which involved an African based bioinformatics group at ILRI (Gardner et al., (2005) *Science*, 1 July 2005: Vol. 309 no. 5731 pp. 134-137.)

**2005** Publication of the *E. ruminantium* genome by African researchers (Collins et al., (2005) *PNAS*, Vol: 102, no. 3, pp. 838-843.)

**2005** Fourth WHO/TDR training course on the genomics and bioinformatics of the Tsetse fly in South Africa (SANBI) involving African researchers.

**2006** The International Bioinformatics Software School, Moroccan Society for Bioinformatics, Morocco.

**2006** Status of bioinformatics training in Africa report was released as an outcome of the meeting with CNRS and African bioinformaticians.

**2006** Fifth and final WHO/TDR bioinformatics training workshop was held in South Africa (SANBI).

**2006** First MSc in Bioinformatics degree was awarded in Nigera.

**2006** ILRI, SLU, EMBNet Introductory bioinformatics course, Kenya (repeat in 2007).

**2007** First ASBCB conference held in Nairobi, Kenya.

**2007** Honours programme in Bioinformatics started at University of Cape Town.

**2007** EBI Ensembl workshop, Kenya

**2008** MSc in Bioinformatics degree program at the Faculty of Sciences, Rabat, Morocco started.

**2008** MSc in Bioinformatics degree program at the National School of Applied Sciences, Tangier Morocco started.

**2008** MSc in Bioinformatics degree program in Tunisia was stopped.

**2008** Second International Bioinformatics Software School IBSS, Morocco.

**2008**  The African Bioinformatics Network (ABioNet) was established.

**2008** ILRI, BeCANet, KARI-TRC, WHO/TDR, Introductory course on tsetse and trypanosomiasis bioinformatics, Kenya.

**2008** University of Rome, (RSG) East Africa, ILRI-BecA Introductory course in Proteomics, Kenya.

**2009** The first edition of NCBI tools and resources training series commenced at Mohamed V University, Rabat, Morocco.

**2009** NIH and Wellcome Trust convened the Frontiers Meeting in Yaoundé, Cameroon.

**2009** First PhD degree in Bioinformatics was awarded in Nigeria.

**2009** A WHO sponsored meeting took place in Abuja, Nigeria.

**2009** Second ASBCB conference was held in Bamako, Mali.

**2009** NBN, South Africa, closed down.

**2009** Third International Bioinformatics Software School IBSS was organized, Morocco

**2009** Introductory and advanved Bioinformatics workshops were organized by Zagazig University, Egypt, repeated in 2011.

**2009** Condensed Bioinformatics (Introduction & Advanced), workshops were organized by Zagazig University, Egypt.

**2010** First MSc degree in bioinformatics was awarded in Morocco.

**2010** CEBIB graduated the first postgraduate student with an MSc in Bioinformatics from a Kenyan University.

**2010** Rhodes University Bioinformatics Research Group (RUBi), South Africa, was established. It became a Unit (Research Unit in Bioinformatics – still abbreviated to RUBi) in 2013.

**2010** H3Africa initiative was launched.

**2010** EBI Tools for Genomics and Proteomics workshop was organized, Morocco.

**2010** Applications of Bioinformatics workshop, Zagazig University, Egypt.

**2010** Advanced international course in translational bioinformatics (29 November to 18 December) was held at Institut Pasteur de Tunis, Tunisia

**2010** The CPGR became the associated node of the EMBNET training network.

**2010** Mini Internship Programme (MIP) started at the CPGR.

**2010** First live online pathway analysis training was provided by IPA and CPGR.

**2011** One-year coursework and dissertation MSc in Bioinformatics program was re-initiated at Rhodes University.

**2011** Third ASBCB conference was held in Cape Town, South Africa.

**2011** ABioNet applied to NIH for funding to form a pan African bioinformatics Network for H3Africa.

**2011** Knowledge Transfer Programme (KTP) for South Africa was established at the CPGR.

**2011** First edition of "Introduction to bioinformatics and medical information" course series commenced at Mohamed V University, Rabat, Morocco.

**2011** Introduction of Bioinformatics workshop, Fayoum university, Egypt.

**2011** Workshop on Biological and Genomics Data Mining (BIOMINING) was given in Morocco.

**2011** EMBO Global Exchange Lecture course 2011: Bioinformatics tools, ressources and applications, Morocco.

**2011** EMBO Global Exchange Lecture course 2011: Next Generation Sequencing for Africa, Kenya.

**2012** H3ABioNet, a pan-African Bioinformatics Network was established.

**2012** MSc in Bioinformatics program commenced at the Future University, Sudan.

**2012** South African Society for Bioinformatics (SASBi) was established.

**2012** First MSc in Bioinformatics degrees were awarded in Mauritius.

**2012** Chipster workshop was given in SA by CPGR/UCT/SANBI.

**2012** EMBO Global Exchange Lecture course 2011: Bioinformatics for microbial Genomics, Morocco.

**2012** Genomics and Molecular Biology Applications for Pathogen Detection and Characterization workshop was held (16 January - 4 February) in Institut Pasteur de Tunis, Tunisia.

**2012** Intensive Bioinformatics Training workshop, Fayoum University, Egypt.

**2012** Basic and advanced bioinformatics workshops, Fayoum University, Egypt.

**2012** Joint ILRI-BecA, UNESCO, Advanced genomics and bioinformatics: Viral/bacterial metagenomics and next generation sequencing workshop, Kenya.

**2013** H3ABioNet Grant Management workshop, University of Cape Town, South Africa.

**2013** H3ABioNet 2-week systems administration workshop University of Pretoria, South Africa.

**2013** H3ABioNet 5-week train the trainer and eBioKits workshop, ICIPE, Kenya

**2013** H3ABioNet Introduction to Bioinformatics using the eBioKit platform, ICIPE, Kenya

**2013** Fourth ASBCB conference held Casablanca, Morocco.

**2013** The re-launching of the Northern African RSG affiliated with the ISCB Student Council, Morocco.

**2013** First Northern African computational biology symposium was held at Mohammed First University, Nador, Morocco.

**2013** Egyptian Center for Bioinformatics and Genomics was established in Egypt.

**2013** First African Affymetrix University was hosted in South Africa by the CPGR.

**2013** First knowledge expert was brought into Africa by KTP.

**2013** First NGS workshop and road show was given by KTP

**2013** South Africa joined SeqaHead COST action.

**2013** Molecular Biochemistry in Health Applications was held in Institut Pasteur de Tunis, Tunisia (7 to 26 January).

**2013** Utilization of bioinformatics and genomics in biological experiments workshop, Zagazig University, Egypt.

**2013** Introduction to Bioinformatics and Programing workshop was given at Zagazig University, Egypt.

**2014** Applications of genomics in public health for infectious diseases of poverty: diagnostics and vector control was held in Institut Pasteur de Tunis, Tunisia (21 to 31 January).

**2014** Principles of Bioinformatics: Sequence Analysis Tools workshop, Ain Shams Center for Genetic Engineering and Biotechnology, Egypt

**2014** H3ABioNet bioinformatics curriculum development workshop held in Gaborone, Botswana.

**2014** H3ABioNet 5-week postgraduate bioinformatics training workshop, Covenant University, Nigeria.

**2014** H3ABioNet Data Management workshop, University of Cape Town, South Africa.

**2014** University of Bamako, Mali, established an MSc Bioinformatics degree program.

**2014** Covenant University, Nigeria, established a 4-year BSc (Hons) Bioinformatics program.
